# Effects of 12 Weeks of Combined Exercise on Heart Rate Variability and Dynamic Pulmonary Function in Obese and Elderly Korean Women

**Published:** 2018-07

**Authors:** Jisu KIM, Hun-Young PARK, Kiwon LIM

**Affiliations:** 1. Physical Activity and Performance Institute (PAPI), Konkuk University, Seoul, Republic of Korea; 2. Dept. of Physical Education, Konkuk University, Seoul, Republic of Korea

**Keywords:** Heart rate variability (HRV), Dynamic pulmonary function (DPF), Combined exercise, Obese elderly women

## Abstract

**Background:**

We investigated whether a combination of aerobic and resistance exercise administered over a period of 12 weeks enhanced heart rate variability (HRV) and dynamic pulmonary function (DPF) in obese and elderly Korean women.

**Methods:**

The study was conducted in 2016 in the Konkuk University (Seoul, Korea). The study participants included 20 older obese women [aged 66.4±0.8years; >30 BMI and >30% in percent body fat]. The subjects were divided into a non-exercise group (n=10, control group; CON) and a combined exercise group (n=10, experimental group; EXP). Total power (TP), low frequency (LF), high frequency (HF), and LH/HF ratio were measured as frequency-domain methods. Salivary cortisol levels were analyzed by ELISA. The participants underwent dynamic pulmonary function (DPF) test.

**Results:**

The EXP group showed a significantly decrease in body weight (*P*=0.002) and % body fat (*P*<0.001) following 12 weeks of combined exercise training. The CON group revealed a significant increase in LF (*P*=0.011), LF/HF ratio (*P*<0.001), salivary cortisol (*P*=0.015) and decrease in HF (*P*=0.003). However, the EXP presented a significant decrease in LF (*P*=0.006) and salivary cortisol (*P*=0.046), and a significantly increase in MVV (*P*<0.001).

**Conclusion:**

Twelve wk of combined aerobic and resistance exercise improves heart rate variability, reducing mental stress in obese older women. In addition, the exercise program was found to be effective in reducing body fat and improving lung function in obese elderly women in East Asian countries with similar body composition and cultural patterns. Therefore, further collaborative research is needed to investigate obese older women in East Asian countries.

## Introduction

Aging of the global population has become a public health concern with an important socioeconomic dimension (1). This issue has been associated with increased obesity rates due to challenges as associated with decreased energy metabolism, sex hormones and muscle mass. Obesity in the elderly population is a challenge not only in the US but also in Europe and East Asian countries (2). Elderly individuals with obesity and overweight manifest an increased risk of premature mortality, cardiovascular disease, cancer, type 2 diabetes, and several other diseases (3, 4). Therefore, anti-aging and anti-obesity studies are a critical component of public health.

In South Korea, obesity rates have increased sharply over the past two to three decades, particularly in older women, rapidly climbing from 22.2% in 1988 to 37.4% in 2015 (5). Even in East Asian countries containing populations with a body composition similar to that of Korea, the rate of elderly obesity is increasing sharply (6). The anti-obesity measures in East Asians need to be investigated because of their similar body composition including height, weight, fat ratio, and muscle mass (7, 8).

However, the majority of obese and aging studies were conducted in populations of European ancestry and United States whereas studies of obese older women in East Asian remain limited (6). Furthermore, most obesity-related studies have focused on abdominal or central obesity, hypertension, insulin resistance, high-triglyceride and low HDL-cholesterol levels, which are associated with metabolic syndrome (9, 10). However, the numerous changes in health-related indicators that occur during the process of obesity and aging need to be investigated. In particular, heart rate variability (HRV) and dynamic lung function (DPF) reduction are indicators of age and obesity (11, 12).

HRV is the physiological variation in time interval between heartbeats. It is measured by the variation in the beat-to-beat interval that non-invasively assesses the activity of the autonomic nervous system (12). According to recent reports, 24-hour Holter monitoring of populations with individuals between 20 and 70 years, revealed an age-related decrease of all parameters, especially parasympathetic cardiac activity due to aging (13). The decreased HRV indicated an abnormal functioning of the autonomic nervous system, and has been associated with mental stress (14). Chronic autonomic activation and/or disturbances may influence several physiological parameters, and increase the risk of cardiovascular disorders (15). Therefore, HRV is a clinically useful tool. In addition, aging results in decreased lung function. To prevent this decline, aerobic exercise is reportedly helpful in improving lung function. Recent reports suggest that prolonged aerobic exercise can improve lung function (16).

Nevertheless, whether prolonged aerobics combined with resistance exercise affects HRV and DPF in obese older women remains unclear. Therefore, the purpose of this study was to investigate the effects of 12 wk of aerobic and resistance exercise on HRV and DPF in Korean obese older women.

## Materials and Methods

### Participants

The study was conducted in 2016 in the Konkuk University (Seoul, Korea). The study participants included 20 obese older women (66.4±0.8 age), who were not exposed to any medication and with a BMI (by Broca’s index) >30 and percent body fat >30%. These women were housewives with low levels of activity who had not performed any kind of exercise over the last 6 months.

The participants provided written informed consent after sufficient explanation of the experiment and an understanding of the possible adverse effects. They were assigned to a no-exercise group (n=10, control group; CON) and a combined exercise group (n=10, experimental group; EXP). There were no significant differences in physical characteristics among groups before exercise intervention (Table 1).

**Table 1:** Participant demographic characteristics

***Variables***	***CON***	***EXP***
Number	10	10
Age (yr)	66.5±0.8	66.3±0.8
Height (cm)	160.7±4.7	160.3±4.8
Weight (kg)	69.5±4.9	69.1±4.6
Free fat mass (kg)	44.5±3.1	44.2±3.0
Body fat (%)	32.0±1.7	31.9±1.7

*Note*. CON = control group, EXP = experimental group

This study was approved by the Institutional Review Board of KyungHee University (KHSIRB-16-016) in Korea.

### Study Design

The study was designed as follows: 2 days of pre-test session (i.e., 2 testing days and 1 day of rest between testing days), 12 wk of combined exercise session, and finally, 2 days of post-test session. The post-test started three days after the last training session. The training session began before three days and the post-test began three days after the last training session.

On the first test day, all participants underwent fasting for more than 10 h and after stabilization, saliva was collected between 7:00 and 9:00 am and body composition measured. On the second test day, the participants underwent fasting for 4 h; heart rate variability (HRV) and dynamic pulmonary function (DPF) were measured in order in the morning.

The participants in EXP performed the following three kinds of combined exercise interventions for 90–120 min: aerobic exercise on a treadmill, aerobic exercise on a bicycle, and elastic resistance exercise. On the other hand, CON did not perform any exercise. All exercise interventions were performed at a constant temperature and humidity (22 ° C, 60%) for a total of 12 wk, three times a week. The maximal heart rate (HRmax) of the participants in EXP aerobic exercise involving a treadmill and a bicycle was calculated using the Karbonen formula (206.9- [0.67 × age]). They performed 60 min of aerobic exercise corresponding to 60–70% of HRmax (treadmill exercise 30 min and bicycle exercise 30 min, respectively). Further, older obese women in EXP performed elastic resistance training sessions involving front squat, incline chest press, seated row, push press, split squat, and pull apart. The participants in EXP performed 3 sets of 10–15 repetitions at an exercise intensity ranging from 6 to 7 on the OMNI-Resistance Exercise Scale of Perceived Exertion (OMNI-RES AM; from 0, extremely easy to 10, extremely hard), which corresponds to exercise intensity levels ranging from 60% to 70% of 1 RM, with rest for 90 s per set. Elastic resistance training sessions were conducted for approximately 30–40 min.

### Measurement

Body composition (i.e., height, weight, fat-free mass, and percent body fat) was measured after fasting for more than 4 h using bioelectrical impedance analysis (BIA).

HRV was measured according to the following procedure. All subjects abstained from coffee, alcohol, and tobacco during the intervention period and HRV was measured after fasting for 4 h, in a room where noise was relatively blocked. Following approximately 10 min of rest, four pads were placed on both wrists and ankles using an HRV meter (LAXTHA, CANS-3000, Korea), and measured under sedentary and sleeping conditions. HRV was analyzed using time-domain methods based on beat-to-beat or normal-to-normal (NN) intervals, and frequency-domain methods assigned to bands of frequency followed by counting the number of NN intervals matching each band. The average of all RR intervals (mean RR), standard deviation of successive differences (SDDN), and root mean square of successive differences (RMSSN) were analyzed as time-domain methods. The total power (TP), low frequency (LF), high frequency (HF), and LH/HF ratio were measured as frequency-domain methods.

For salivary cortisol measurement, all participants underwent fasting for more than 10 h before the test, and abstained from brushing teeth. Further, the participants rinsed their mouth with cold water 10 min before collecting the saliva, and refrained from eating or salivating. The collected frozen saliva was analyzed via ELISA using the ER HS SALIVARY CORTISOL kit (Salimetrics, Pennsylvania, USA).

For DPF test, the participants were aspirated to the maximum and breathed to the end as quickly, and as efficiently as possible. This process was repeated 2–3 times and the highest value was used as the result. Forced vital capacity (FVC), forced expiratory volume in 1 sec (FEV_1.0_), and percent of forced expiratory volume in 1 sec (FEV_1.0_/FVC ration) were measured. The maximal voluntary ventilation (MVV) obtained by repeated breathing and inspiration for 12 sec was used to evaluate DPF.

### Statistical analysis

All statistical analyses were conducted using SPSS 23.0 (IBM Corp., Armonk, NY, USA) for Windows. Data are presented as mean ± standard deviation. The sample size was calculated to improve the statically power to more than 95% using G-power. Normality of distribution of all outcome variables was verified using a Kolmogorov-Smirnov test. A two-way analysis (time × group) of variance with repeated measures of the “time” factor was used to analyze the effects of training programs on each dependent variable. If a significant interaction effect was found, a post-hoc Bonferroni test was used to identify within-group change over time. Additionally, a paired *t-*test was used to compare post-training compared with pre-training values of dependent variables in each group separately. The a priori level of significance was set at 0.05.

## Results

### Body Composition

As shown in Table 2, there was a significant interaction in all body composition parameters including body weight (F=14.044, *P*=0.001), FFM (F=10.608, *P*=0.004), and %body fat (F=240.652, *P*<0.001). The post-hoc analyses showed a significant decrease in FFM (*P*=0.013) and increase in %body fat (*P*<0.001) in the CON group. However, the EXP presented a significant decrease in body weight (*P*=0.002) and %body fat (*P*< 0.001) after 12 wk of combined exercise training.

**Table 2:** Changes in body composition parameters by training in CON and EXP before and after training

***Variables***	***Groups***	***Before training***	***After training***	***ANOVA (F-value)***	
Weight (kg)	CON	69.5±4.9	70.2±4.9	Time	2.375
	EXP	69.1±4.6	67.5±4.4†	Group	0.566
Free fat mass (kg)	CON	44.5±3.1	43.5±3.0†	Time×Group	14.044*
				Time	2.866
				Group	0.079
	EXP	44.2±3.0	44.5±2.9	Time×Group	10.608*
Body fat (%)	CON	32.0±1.7	33.7±1.9†	Time	1.549
				Group	6.677*
	EXP	31.9±1.7	29.9±1.5†	Time×Group	240.652*

*Note*. CON = control group, EXP = experimental group (Mean±S.D.)

* Significant (*p*<.05) interaction or main effect

† *P*<.05 vs. before training

### Heart Rate Variability and Salivary Cortisol

Table 3 displays data for stress-induced HRV before and after 12 wk of combined exercise training in both groups.

**Table 3:** Changes in HRV and salivary cortisol by training in CON and EXP before and after training

***Variables***	***Groups***	***Before training***	***After training***	***ANOVA (F-value)***	
Mean RR (ms)	CON	855.8±99.0	828.5±91.0	Time	0.005
				Group	0.224
	EXP	841.5±58.7	871.7±76.8	Time×Group	1.894
SDDN (ms)	CON	37.3±16.2	35.6±9.7	Time	0.088
				Group	0.270
	EXP	36.6±8.2	40.1±6.3	Time×Group	0.740
RMSSD (ms)	CON	18.0±4.1	20.2±5.1	Time	14.335*
				Group	0.370
	EXP	17.6±3.8	22.7±4.6	Time×Group	2.417
TP (ms^2^)	CON	7.16±0.57	6.96±0.35	Time	0.006
				Group	0.079
	EXP	7.03±0.76	7.21±0.66	Time×Group	1.437
LF (Hz)	CON	5.46±0.73	5.95±0.51†	Time	0.337
				Group	0.848
	EXP	5.67±0.45	5.29±0.67†	Time×Group	21.450*
HF (Hz)	CON	6.11±1.34	5.59±1.13†	Time	2.573
				Group	0.524
	EXP	5.44±0.67	5.56±1.15	Time×Group	6.476*
LF/HF ratio	CON	0.93±0.23	1.10±0.23†	Time	3.413
				Group	0.010
	EXP	1.06±0.16	1.00±0.26	Time×Group	17.768*
Salivary Cortisol (ug/dL)	CON	0.30±0.08	0.41±0.12†	Time	4.440*
				Group	10.077*
	EXP	0.26±0.08	0.23±0.06	Time×Group	12.452*

*Note*. HRV = heart rate variability, Mean RR = average of all RR interval, SDDN = standard deviation of the RR interval, RMSSD = the square roof of the mean squared difference of successive RR interval, TP = total power, LF = low frequency, HF = high frequency, CON = control group, EXP = experimental group. //

* Significant (*P*<.05) interaction or main effect //

† *P*<.05 vs. before training

No significant interaction was observed in mean RR, SDDN, and RMSSD related to time-domain methods. However, there was a significant interaction between HF (F=21.450, *P*<0.001), LF (F=6.476, *P*=0.020), and LF/HF ratio (F=17.768, *P*=0.001) using frequency-domain methods. In addition, salivary cortisol showed a significant interaction (F=12.452, *P*=0.002). Post-hoc analyses revealed significant increases in LF (*P*=0.011), LF/HF ratio (*P*<0.001), salivary cortisol (*P*=0.015) and decrease in HF (*P*=0.003) in the CON group. However, the EXP presented a significant decrease in LF (*P*=0.006) and salivary cortisol (*P*=0.046).

### Dynamic Pulmonary Function

Data for DPF before and after the combined exercise training in both groups are shown in Table 4. There was a significant interaction with FVC (F=9.382, *P*=0.007) and MVV (F=32.126, *P*<0.001). Post-hoc analyses showed a significant decrease in FVC (*P*=0.011) of the CON group, and a significant increase in MVV of the EXP group (*P*<0.001).

**Table 4:** Changes in DFP by training in CON and EXP group before and after training

***Variables***	***Groups***	***Before training***	***After training***	***ANOVA (F-value)***		
				**Time**	**Group**	**Time×Group**
FVC (L)	CON	2.93±0.25	2.87±0.22	2.292	0.154	9.382*
	EXP	2.85±0.26	2.88±0.24			
FEV_1.0_ (L)	CON	2.19±0.19	2.20±0.17	1.036	0.549	0.100
	EXP	2.12±0.20	2.14±0.18			
FEV_1.0_/FVC ratio	CON	75.1±1.4	74.9±1.4	0.225	0.020	0.273
	EXP	75.1±1.4	75.1±1.2			
MVV (L)	CON	75.5±3.0	75.0±2.5	14.394*	0.893	32.126*
	EXP	75.4±2.6	77.4±2.9^†^			

*Note*. DFP = dynamic pulmonary function, FVC = forced ventilator capacity, FEV1.0 = force expiratory volume in 1 second, MVV = maximum voluntary ventilation, CON = control group, EXP = experimental group.

* Significant (*P*<.05) interaction or main effect

† *P*<.05 vs. before training

## Discussion

This study investigated HRV and DPF following long-term aerobic and resistance exercise in obese elderly women. Combined aerobic and resistance exercises are known to effectively break down body fat. It is known that aerobic and resistance exercise increase energy expenditure and burn fat efficiently. Ho et al. (2010) reported that combined exercise increases energy expenditure, promotes fat oxidation, and induces muscle synthesis, thereby increasing muscle mass (17). However, the results of the study showed no variation in muscle mass of obese elderly women even after long-term resistance exercise (18). The main reason for this is known to be the decline in sex hormones due to aging (19). HRV testing is an effective method for quantitative evaluation of the activity and balance of the autonomic nervous system (12). HRV test is noninvasive, and the test method is relatively simple. The test results can be obtained promptly by computer analysis (20). Further, the activity of the autonomic nervous system can be measured objectively and easily (14). Changes in the activity of the autonomic nervous system and balance due to stress enable the diagnosis of stress-related diseases. Therefore, the HRV test is considered as a useful tool to objectively evaluate the psychological-emotional state of an individual (21).

The results of 12 wk of combined exercise in obese elderly women showed that the frequency domain analysis of HF was significantly higher than that of the control group. Recent reports suggest that depression, panic disorder, and anxiety affect the autonomic nervous system by mainly reducing the activity of the parasympathetic nervous system. HF has been reported to decrease in emotional states such as sustained stress, anxiety, and fear associated with decreased parasympathetic activity (22). LF showed a tendency to decrease compared with the control group. Previous studies have shown that LF is associated with mental stress, and that LF is reduced in fatigue and has a significant positive correlation with depression and anger (23). LF is also known to be elevated in patients with migraine following hyperactivity of the sympathetic nervous system (20). LF/HF ratio reflects the overall balance of the autonomic nervous system. High LF/HF suggests a relative increase in the activity of sympathetic nervous system or suppression of the parasympathetic activity (12). The LF/HF ratio increases when sympathetic activity is relatively dominant, such as anxiety, fear, tension, distraction, and awakening (13). Conversely, LF/HF values are reported to be lower when parasympathetic activity is dominant, such as helplessness, nervous breakdown, depression, and low awakening. Our results are consistent with recent studies showing that continuous exercise increases HRV and reduces stress. A previous report suggested that eight weeks of moderate-intensity exercise (at 50% VO2max) of 44 min performed 3 to 4 times each week increases HRV in older women (24).

Cortisol, a type of hormone secreted during stress, is also known to promote senescence. As a result of 12 wk of combined exercise, salivary cortisol levels were found to be significantly decreased, consistent with the findings of previous studies showing that continuous exercise reduces cortisol levels. Forty two elderly subjects showed decreased cortisol level following 12 wk of aerobic and anaerobic exercise. Therefore, it was suggested that the cortisol reduction of through exercise improves quality of life (25).

Lungs utilize oxygen for metabolism and release carbon dioxide from our bodies. Defective lung function causes various problems (26). Aging is an important variable that has a statistically significant correlation with pulmonary function (27). Following 12 wk of combined exercise, the improved FVC and MVV was related to the increase in oxygen uptake (28). Taken together, these results suggest that 12 wk of combined exercise reduce body weight and fat content as well as improve HRV and lung function.

## Conclusion

Twelve weeks of aerobic and resistance exercise improves HRV, and reduces mental stress in obese older women. In addition, the exercise program was found to be effective in reducing body fat and improving lung function in obese elderly women in East Asian countries with similar body composition and lifestyle. Therefore, further collaborative research involving obese older women in East Asian countries is needed.

## Ethical considerations

Ethical issues (Including plagiarism, informed consent, misconduct, data fabrication and/or falsification, double publication and/or submission, redundancy, etc.) have been completely observed by the authors.
